# Seroepidemiologic Effects of Influenza A(H1N1)pdm09 in Australia, New Zealand, and Singapore

**DOI:** 10.3201/eid1901.111643

**Published:** 2013-01

**Authors:** James M. Trauer, Don Bandaranayake, Robert Booy, Mark I. Chen, Michelle Cretikos, Gary K. Dowse, Dominic E. Dwyer, Michael E. Greenberg, Q. Sue Huang, Gulam Khandaker, Jen Kok, Karen L. Laurie, Vernon J. Lee, Jodie McVernon, Scott Walter, Peter G. Markey, for the Australia, New Zealand and Singapore Pandemic Serosurveillance Study Group

**Affiliations:** Author affiliations: Melbourne Sleep Disorders Centre, East Melbourne, Victoria, Australia (J.M. Trauer);; Environmental Science and Research, Wallaceville, New Zealand (D. Bandaranayake, Q.S. Huang); National Centre for Immunisation Research and Surveillance, Westmead, New South Wales, Australia (R. Booy, G. Khandaker); National University Health System, Singapore (M.I. Chen); University of Sydney, Sydney, New South Wales, Australia (M. Cretikos);; Communicable Disease Control Directorate, Shenton Park, Western Australia, Australia (G.K. Dowse);; Centre for Infectious Diseases and Microbiology, Westmead (D.E. Dwyer, J. Kok);; CSL Limited, Parkville, Victoria, Australia (M.E. Greenberg);; World Health Organization Collaborating Centre for Reference and Research in Influenza, North Melbourne, Victoria, Australia (K.L. Laurie);; World Health Organization, Geneva, Switzerland (V.J. Lee);; Melbourne School of Population Health, Parkville (J. McVernon); Centre for Epidemiology and Research, North Sydney, New South Wales, Australia (S. Walter; Centre for Disease Control, Tiwi, Northern Territory, Australia (P.G. Markey)

**Keywords:** Influenza A virus, H1N1 subtype, serology, Influenza, human, population groups, viruses, Australia, New Zealand, Singapore, pandemic, influenza A(H1N1)pdm09

## Abstract

To estimate population attack rates of influenza A(H1N1)pdm2009 in the Southern Hemisphere during June–August 2009, we conducted several serologic studies. We pooled individual-level data from studies using hemagglutination inhibition assays performed in Australia, New Zealand, and Singapore. We determined seropositive proportions (titer >40) for each study region by age-group and sex in pre- and postpandemic phases, as defined by jurisdictional notification data. After exclusions, the pooled database consisted of, 4,414 prepandemic assays and 7,715 postpandemic assays. In the prepandemic phase, older age groups showed greater seropositive proportions, with age-standardized, community-based proportions ranging from 3.5% in Singapore to 11.9% in New Zealand. In the postpandemic phase, seropositive proportions ranged from 17.5% in Singapore to 30.8% in New Zealand, with highest proportions seen in school-aged children. Pregnancy and residential care were associated with lower postpandemic seropositivity, whereas Aboriginal and Torres Strait Islander Australians and Pacific Peoples of New Zealand had greater postpandemic seropositivity.

## Abstract 2

Temperate regions, school-aged children, and native peoples were particularly susceptible to the first wave of a novel influenza strain.

Australia, New Zealand (NZ), and Singapore all experience regular influenza seasons that coincide with winter in the Southern Hemisphere. After pandemic influenza A(H1N1) 2009 (A[H1N1]pdm09) emerged during spring in North America (*1*), influenza notifications and other markers of influenza activity peaked in Australia, NZ, and Singapore during July 2009 (*2*–*4*). The 3 countries continued to experience the circulation of an influenza strain closely related to the original virus until at least the following winter (*5*).

Most influenza surveillance systems are passive, laboratory-based systems that capture only symptomatic patients who seek medical advice and are then appropriately tested and case notifications sent. Therefore, these systems are likely to underestimate the true attack rate. Measurement of antibodies against A(H1N1)pdm09 can be used to assess the extent of population exposure to the virus (*6*). The emergence of a novel influenza virus provided a unique opportunity to study the behavior of influenza viruses to better understand their differential effects across various population groups.

Standardization of epidemiologic and serologic techniques across our region enabled more direct comparison of the effects of pandemic influenza on the different populations studied. Three of the countries in our region performed such studies, with publications originating from Australia (*7*–*15*), NZ (*16*), and Singapore (*17*). We pooled individual-level serologic data from studies that used the hemagglutination inhibition (HI) assay to describe the effects of the 2009 winter influenza pandemic in the Southern Hemisphere.

## Methods

### Identification of Studies

A working group for pandemic influenza serologic studies was formed with assistance from the Australian Seasonal Influenza Surveillance Strategy Working Group. The aims of this group included standardization of methods to facilitate analysis of pandemic serosurveillance research undertaken across Australia. The group convened its first teleconference on September 29, 2009, and continued to meet regularly as the studies were performed. Through this group and its contacts, 11 teams of researchers were identified who had performed serologic studies. After expressions of interest from researchers in Singapore (which lies just north of the equator but has a mid-year peak in influenza notifications) and NZ, 2 additional groups were identified.

Additionally, we searched Embase and PubMed for the period January 2009 to April 2011 using a combination of database-specific controlled vocabulary and general free text terms, including the following: “influenza A virus, H1N1 subtype,” “seroepidemiologic studies,” “influenza,” “seroepidemiology,” “serosurvey,” and geographic terms for regions of the Southern Hemisphere. No further studies were identified by using these search strategies.

### Inclusion Criteria

Studies were eligible for inclusion if they assessed serologic immunity against A(H1N1)pdm09 by HI assay across a population group in the Southern Hemisphere or Singapore. Studies were eligible if collected before vaccination programs against the virus commenced or if strategies were in place to allow for vaccine effect. Investigators from contributing studies provided HI assay titers, collection date, age, and geographic location at the individual level. The Figure shows the study profile.

### Pandemic Phases

We defined the study region as NZ, Singapore, or Australian state or territory. Because the definition of pandemic phases varied between included studies, we defined pandemic phases using generally more stringent criteria than those used in contributing studies. Prepandemic specimens were defined as those collected before the first notified case in the corresponding region. Postpandemic phases were defined using notification data from NZ and the Australian Government Department of Health and Ageing by week and region. For these countries, we defined postpandemic specimens as those collected at least 2 weeks after the date on which 90% of 2009 laboratory notifications had occurred for the region. In Singapore, continuing pandemic activity was noted through late 2009. Because the adult studies from Singapore were repeated collections from prospective cohorts, the latest collection was used for estimates of postpandemic immunity, generally from October 2009. The postpandemic collection from children in Singapore was from September 1, 2009, to June 2, 2010, and all of these specimens were included as postpandemic. Specimens that did not meet criteria for prepandemic or postpandemic were defined as intrapandemic and excluded from further analysis.

### Statistical Analysis

Most studies were performed as cross-sectional or analysis of continuous prospective collections of available specimens collected for other purposes. Studies that used a purposive sampling technique were analyzed in the same way as those that used convenience collections. In the case of cohort collections and clinical trials, pre- and postpandemic assays from the same person were delinked and analyzed independently for consistency with other study techniques. For clinical trials, preintervention data from the intervention group and all data from the control group were included, whereas postintervention data from the treatment group were excluded. One study (M) used a postpandemic, cross-sectional design with retrospective assessment of prepandemic titers for those specimens found to be seropositive. For this study, only the postpandemic collection was included.

All studies used 2-fold serial dilutions from an initial dilution of 1:10 to determine titers. A titer of >40 was used to define seropositivity because all included studies used this cutoff value. Two studies (L, O) reported the geometric mean of 3 assays for each specimen, and for these studies, geometric mean titers of >40 were used to define seropositivity. Seropositive proportions are expressed as the proportion of reciprocal titers >40, with 95% CIs. Seropositive proportions are only reported for groups represented by >20 specimens. For comparability, age-standardized assessments of the proportion seropositive were calculated, weighted by 5-year age brackets to a reference population (Australian population on June 1, 2009) (*18*). Attack rates are calculated (for populations for which pre- and postpandemic seropositive proportions were available) as the difference in proportions of the immune population between pre- and postpandemic groups, age-standardized to the same reference population.

Using data from the 11 community-based studies, we performed multivariate logistic regression for the outcome of seropositivity in pre- and postpandemic phases. Exposure variables included in the model consisted of sex, age group, and study region because no other variables were consistently available across datasets.

To quantify the effect of study methods and the presence of potential risk factors on seropositivity, we compared pairs of studies performed in similar populations, using multivariate logistic regression, on the outcome of seropositivity. Data from the reference study were included along with data from a study of persons with the most similar characteristics. Exposure variables consisted of age, sex, and the binary variable of comparison group versus reference group. Analyses were restricted to specimens taken from patients during the same pandemic phase with comparable demographic characteristics (age, region, and population). Data management and statistical analysis were carried out with Stata 11.0 (StataCorp LP, College Station, TX, USA).

## Results

Datasets were received from 11 groups of investigators, consisting of data from 10 published and 3 unpublished studies. Data were received from NZ, Singapore, and New South Wales (NSW), the Northern Territory (NT), Queensland, Tasmania, Victoria, and Western Australia in Australia. Datasets are listed by study design and population, with pandemic phases referring to investigators’ definitions, which resulted in 19 datasets for analysis. Study designs consisted of 4 prospective cohorts (E–H), 3 randomized controlled trials (L, O, R), 2 prepandemic cross-sectional studies (A, D), 1 retrospective cohort study (M), and 6 unpaired pre-and postpandemic cross-sectional studies (I, J, K, N, P, S). Eleven datasets were community based, whereas 8 were from groups with potential risk factors.

Laboratory techniques common to all studies included HI assay, per inclusion criteria, and use of egg-grown, β-propiolactone–inactivated A/California/07/2009 reference virus as the antigen source. All studies provided titers and patient’s age in years for each assay, and all Australian studies provided geographic data to at least state/territory level. Table 1 illustrates study characteristics that differed between included studies, with studies differing by design, enrolment criteria, specimen type, and specific HI method. All studies but 2 (I, K) provided data on patient’s sex for all assays. All studies attempted to avoid contamination of specimens from vaccination effect, but approaches used to achieve this differed.

**Table 1:** Characteristics of collections included in database to estimate population attach rates of influenza A (H1N1) 2009 in the Southern Hemisphere, winter 2009*

Code (ref. no.)	Study design	No. assays by redefined phase	Population	Age range, y†	Study exclusions	Enrolment	Region	Spec-imen type	Testing lab-oratory	RBC species	Control serum specimen	Monovalent pandemic vaccine effect	Notes and exclusions for analysis
A (*16*)	Pre cross-section	524 pre	Outpatients	1–99	None	Opportunistic from stored specimens	NZ	Serum	ESR	Guinea pig	Human and ferret	Not applicable	None
B (*16*)	Post cross-section	1,147 post	Primary care patients	1–89	None	Active recruitment of registered GP patients	NZ	Serum	ESR	Guinea pig	Human and ferret	Collection prior to vaccination program	None
C (*16*)	Post cross-section	532 post	Health care workers‡	21–109	None	Active recruitment of hospital and clinic staff	NZ	Serum	ESR	Guinea pig	Human and ferret	Collection prior to vaccination program	None
D (*7*)	Pre cross-section	152 pre	Residents of aged-care facilities‡	59–100	None	Outbreak investigations of non-H1N1 viruses	NSW	Serum	CIDMLS	Human, O negative	Human	Not applicable	None
E (*17*)	Prospective cohort (pre and post collections)	788 pre	Community residents	21–74	None	Sub-cohort of existing cohort collection	Singapore	Serum	WHO-CC	Turkey	Human and ferret	Collection prior to vaccination program	None
		671 intra											
F (*17*)		689 post											
	Prospective cohort (pre and post collections)	1 pre	Healthcare workers‡	20–67	None	Email and word of mouth staff recruitment at hospital	Singapore	Serum	WHO-CC	Turkey	Human and ferret	Collection prior to vaccination program	None
		1,138 intra											
		391 post											
G (*17*)	Prospective cohort (pre and post collections)	300 intra	Staff and residents of long-term care facilities‡	19–109	None	Active recruitment by invitation	Singapore	Serum	WHO-CC	Turkey	Human and ferret	Collection prior to vaccination program	None
		250 post											
H (*17*)	Prospective cohort (pre and post collections)	1,915 intra	Military personnel‡	18–62	None	Active recruitment by invitation	Singapore	Serum	WHO-CC	Turkey	Human and ferret	Collection prior to vaccination program	None
		637 post											
I (*8*)	Pre and post cross-sections	447 pre	Community residents	0–19	Respiratory infection indication for testing	Opportunistic from pathology laboratory	WA	Serum	WHO-CC	Turkey	Human and ferret	Collection prior to vaccination program	Gender unavailable
		221 intra											
		229 post											
J (*8*)	Pre and post cross-sections	201 pre	Pregnant women‡	21–45	Respiratory infection indication for testing	Opportunistic from pathology laboratory	WA	Serum	WHO-CC	Turkey	Human and ferret	Collection prior to vaccination program	None
		170 intra											
		116 post											
K (*9*)	Pre and post- cross-sections	474 pre	Outpatients	0–100	Influenza serologic testing	Opportunistic from pathology laboratories	NSW	Serum or lithium-heparin plasma	CIDMLS	Human, O negative	Human	Collection prior to vaccination program	Gender unavailable for 164 pre-pandemic assays
		750 intra											
		497 post											
L (*10*)	RCT of pandemic vaccine (pre-vaccine collection)	166 intra	Healthy adults	.	Pregnancy	Active recruitment of volunteers	Adelaide	Serum	Focus	Turkey	Human	Collection prior to vaccination program	Postvaccinationassays excluded
M (*11*)	Postpandemic cross-section with retrospective assessment of seroconversion	125 intra	Persons infected with HIV‡	19–77	None	Opportunistic testing of specimens submitted for HIV load monitoring	NSW	Plasma	CIDMLS	Human O-negative	Human	Collection prior to vaccination program	Retrospective assays excluded
		74 post											
N (*12*)	Pre and post cross-sections	404 pre	Blood donors	16–78	None	Opportunistic collection from donated blood units	Cairns, Townsville, Brisbane, Hobart, Melbourne, Newcastle, Perth, Sydney	Plasma	WHO-CC	Turkey	Human and ferret	Vaccination status checked if titer raised	None
		92 intra											
		779 post											
O (*13*)	RCT of pandemic vaccine (pre-vaccine collection)	290 intra	Community volunteers	0–8	Gestational age <36 wk, investigational vaccine	Active recruitment through tertiary hospitals	Adelaide, Brisbane, Melbourne, Perth, Sydney	Serum	Focus	Turkey	Human	Collection prior to vaccination program	Postvaccination assays excluded
		73 post											
P (*14*)	Pre and post cross-sections	443 pre	Outpatients	0–97	None	Opportunistic from pathology laboratory	NT	Serum	WHO-CC	Turkey	Human and ferret	Collection prior to vaccination program	None
		2 intra											
		1,689 post											
Q (unpub.)	Post cross-sections	65 post	Hemo-dialysis patients‡	43–88	None	Prevaccination blood sample	NSW	Serum	CIDMLS	Human, O negative	Human	Collection prior to individual vaccination	None
R (unpub.)	RCT of interferon (pre- and postintervention collections)	64 pre	Community volunteers	20–74	Chronic respiratory conditions	Active recruitment (email and newspaper)	Perth	Serum	WHO-CC	Turkey	Human and ferret	Patients receiving vaccine excluded	Postintervention assays in active treatment arm excluded
		102 intra											
		87 post											
S (unpub.)	Pre and post cross-sections	944 pre	Community residents	0–18	None	National pediatric serosurveillance studies	Singapore	Serum	WHO-CC	Turkey	Human and ferret	Low vaccine uptake (≈1%–2%) in population at time of collection (*19*)	Postvaccine assays excluded
		32 intra											
		460 post											

*RBC, red blood cells; pre, prepandemic phase; intra, intrapandemic phase; post, postpandemic phase; ESR, World Health Organization National Influenza Centre, Environmental Science and Research, New Zealand; CIDMLS, Centre for Infectious Diseases and Microbiology Laboratory Services, Institute of Clinical Pathology and Medical Research, Westmead Hospital, Sydney, Australia; WHO-CC, World Health Organization Collaborating Centre for Reference and Research on Influenza, North Melbourne, Australia; Focus = Focus Diagnostics, California, USA; RCT, randomized controlled trial; GP, general practitioner; NZ, New Zealand, NSW, New South Wales; WA, Western Australia; NT, Northern Territory. †Age range for specimens included in pre or postpandemic phases. ‡Defined as risk groups for analysis.

Received data consisted of 18,279 individual specimens, of which 18,131 assays (from 14,036 persons) were eligible for analysis, whereas 148 did not meet inclusion criteria. Samples were reclassified as prepandemic (4,414), intrapandemic (6,002), or postpandemic (7,715), according to the criteria described, with intrapandemic assays excluded from further analysis (Figure). The timing of the pandemic phases and the sample taking is summarized in the Technical Appendix. Of the assays eligible for analysis in the pre- or postpandemic groups, 125 prepandemic specimens and 2,065 postpandemic specimens were from risk groups, while the remainder were from community-based datasets.

**Figure:**
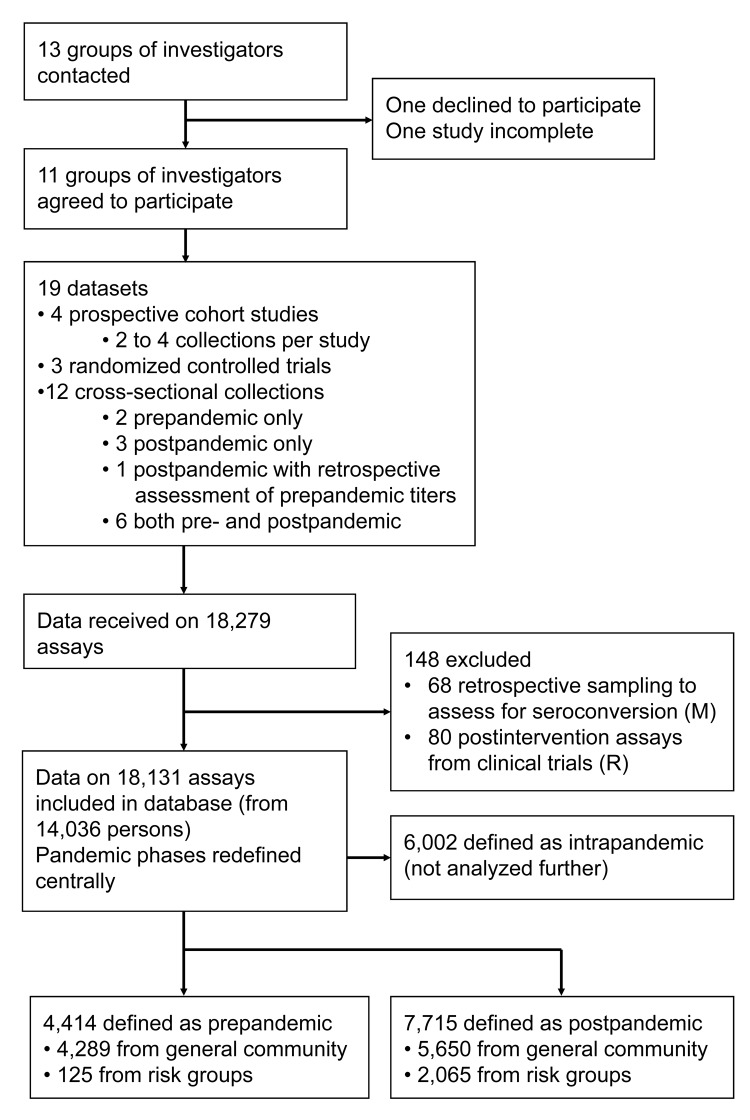
Flow chart showing profile of serologic studies to estimate attack rates of influenza A (H1N1) pandemic 2009 in the Southern Hemisphere during winter 2009.

Tables 2 and 3 show seropositive proportions in the pre- and postpandemic periods. Overall, the age-standardized prepandemic seropositive proportion was 9.4%, with regional estimates of 10.6% in Australia, 11.9% in NZ, and 3.5% in Singapore. Higher levels of immunity were seen with increasing age, with only 1 of 5 studies (A) of children finding evidence of preexisting immunity in age group 0–4 years, whereas markedly higher seropositive proportions were seen in those >75 years of age.

**Table 2:** Prepandemic seropositive proportions by country, region, and risk group of influenza A (H1N1) 2009 in the Southern Hemisphere, winter 2009*

Code	Pop.	Age groups, y							Sex			Overall	
		0–4	5–14	15–34	35–54	55–74	>75		F	M		Raw	Age-stand.
A, E, I, N, P, R, S	Overall	1.4	2.7	12.0	4.8	11.5	47.4		7.8	8.5		8.4	9.4
I, N, P, R	AU	0	1.6	12.2	7.9	12.3	48.8		11.7	9.3		11.0	10.6
A	NZ	7.0	14.9	8.4	5.3	20.1	33.3		9.2	16.5		12.0	11.9
E & S	Sing	0	1.6	12.7	2.0	1.4			2.2	3.3		4.7	3.5
K	NSW	0	5.1	14.2	4.4	24.6	51.9		15.1	9.5		19.6	11.9
P	NT	0	0	4.4	8.7	8.2	26.7		6.5	8.5		7.4	6.9
N	QLD			12.3	11.3	14.4			15.0	10.6		12.6	
I, R	WA	0	0	15.7	0	7.7			12.5	4.2		4.7	9.8
Risk group collection													
D	NSW res. care					28.0	50.5		55.8	23.7		46.0	

*Pop., population; age-stand., age-standardized; AU, Australia; NZ, New Zealand; Sing, Singapore; NSW, New South Wales; QLD, Queensland; NT, Northern Territory; WA, Western Australia; res., residential. Blank cells indicate no data.

**Table 3:** Postpandemic seropositive proportions by country, region, and risk group of influenza A (H1N1) 2009 in the Southern Hemisphere, winter 2009, with age-characterized AR*

Code	Pop.	Age groups, y							Sex			Overall		Age stand. AR
		0–4	5–14	15–34	35–54	55–74	≥75		F	M		Raw	Age stand.	
A, B, E, I, K, N, O, P, R, S	Overall	27.6	34.3	30.5	16.8	18.0	23.3		23.0	22.3		23.8	24.3	14.9
I, K, N, O, P, R	AU	24.0	32.2	29.8	17.8	18.8	17.0		23.3	21.2		23.1	23.7	13.1
B	NZ	37.2	46.3	38.1	22.3	20.1	35.8		30.5	30.1		30.3	30.8	19.0
E, S	Sing.	24.5	29.6	17.2	11.0	6.8			10.7	13.4		19.2	17.5	14.0
K, N	NSW	17.3	18.4	37.8	19.3	18.8	21.6		25.5	24.2		26.2	27.2	15.3
P	NT	16.7	37.2	22.0	18.1	16.3	14.3		20.5	18.3		19.5	21.8	15.0
N	QLD			29.6	9.3	14.8			19.2	18.0		18.5		
N	Tas			35.9	28.9	26.7			35.6	24.5		30.6		
N, O	Vic	36.1		30.8	12.5	21.4			31.3	13.6		21.5		
I, N, R	WA	24.0	39.5	31.6	18.2	34.3			27.4	27.5		31.4	30.3	20.5
Risk group collections														
M	NSW, HIV+				29.5	30.4				35.6		28.4		
Q	NSW, hemo.					21.7	25.0		20.8	22.0		21.5		
C	NZ, HCWs			31.3	23.7	27.6	33.3		26.3	28.2		26.7		
F	Sing. HCWs			11.0	6.8	11.1			10.1	6.3		9.5		
G	Sing. res. care			4.3	2.7	6.8	11.4		4.9	9.4		6.8		
H	Sing. military			35.7	3.4					34.5		33.9		
J	WA, preg. women			13.3	19.2				14.7			14.7		
P	NT, indig.		37.5	28.4	28.1	32.9			28.4	30.9		29.5	29.8	22.1
B	NZ, Maori			42.3	26.2	20.6			39.4	28.0		34.3		
B	NZ, Pacific People	56.0	55.6	53.1	39.5	24.3			43.6	45.1		43.7		
Overall attack rates, community-based studies														
A, B, E, I, K, N, O, P, R, S	Overall	26.2	31.6	18.5	12.1	6.4	–24.1		15.2	13.7		15.3	14.9	
Overall geometric mean titers, community-based studies														
A, E, I, N, P, R, S	Pre	6.03	5.86	8.63	6.97	8.57	24.13		15.2	13.7		15.3	14.9	
A, B, E, I, K, N, O, P, R, S	Post	15.42	16.87	16.09	10.10	11.29	14.74		15.2	13.7		15.3	14.9	

*Results are expressed as % (95% CI). AR, attack rate; Pop., population; Age-stand, age-standardized; AU, Australia; NZ, New Zealand; Sing., Singapore; NSW, New South Wales; NT, Northern Territory; QLD, Queensland; Tas, Tasmania; Vic, Victoria; WA, Western Australia; HCWs, health care workers; hemo., hemodialysis; res., residential; preg., pregnant; indig., indigenous; pre, prepandemic; post, postpandemic. Blank cells indicate no data.

In the postpandemic period, the age-standardized seropositive proportion was 24.3%, giving an attack rate of 14.9% (Table 3). Attack rates by country were 13.1% for Australia, 19.0% for NZ, and 14.0% for Singapore. For all regions in which children 5–14 years of age were assessed, this age group had the highest levels of postpandemic seropositivity, except for NSW, in which those 15–34 years of age showed the greatest seropositivity. Among risk groups, unweighted seropositive proportions were 28.4% in HIV-positive persons in NSW, 21.5% in hemodialysis patients in NSW, 26.7% in NZ health care workers, 9.5% in Singaporean health care workers, 33.9% in Singaporean military personnel, 6.8% in staff and residents of Singaporean residential care facilities, 14.7% in pregnant women in WA, 29.5% in indigenous residents of the NT, 34.3% in Maori in NZ, and 43.7% in Pacific Peoples in NZ.

Logistic regression performed in assays from postpandemic, community-based collections showed that the age groups 5–14 years and 15–34 years, as well as residence in NZ, were associated with increased seropositivity. Negative effects were seen for older age groups and those with residence in Singapore (Table 4).

**Table 4:** Multivariate logistic regression models on outcome of pre- and postpandemic seropositivity in community-based studies of influenza A (H1N1) 2009 in the Southern Hemisphere with exposure variables of region, age group, and sex, winter 2009*

Exposure variable	Prepandemic phase, n = 4,289			Postpandemic phase, n = 5,650	
	Odds ratio (95% CI)	p value		Odds ratio (95% CI)	p value
Region					
New South Wales†	1			1	
New Zealand	1.18 (0.77–1.80)	0.45		1.44 (1.17–1.79)	0.001
Northern Territory	0.62 (0.38–1.02)	0.06		0.82 (0.66–1.01)	0.06
Queensland	1.25 (0.79–1.98)	0.34		0.78 (0.53–1.14)	0.20
Singapore	0.40 (0.27–0.61)	<0.001		0.56 (0.43–0.74)	<0.001
Tasmania				1.50 (0.95–2.35)	0.08
Victoria				1.37 (0.94–2.01)	0.11
Western Australia	0.41 (0.25–0.67)	<0.001		1.04 (0.76–1.43)	0.79
Age group, y					
0–4†	1			1	
5–14	2.34 (0.92–5.92)	0.07		1.60 (1.24–2.06)	<0.001
15–34	13.70 (5.85–32.07)	<0.001		1.50 (1.18–1.91)	0.001
35–54	6.24 (2.50–15.57)	<0.001		0.75 (0.58–0.98)	0.04
55–74	14.60 (5.98–35.62)	<0.001		0.73 (0.56–0.95)	0.02
≥75	47.43 (18.58–121.08)	<0.001		0.95 (0.64–1.41)	0.80
Sex					
F†	1			1	
M	0.99 (0.73–1.34)	0.96		0.97 (0.85–1.24)	0.70
Unknown	2.44 (1.74–3.42)	<0.001		1.67 (1.25–2.24)	0.001

*Blank cells indicate no data. The 2 regression models are displayed vertically. †Reference category.

In 2 instances, the same demographic group was assessed by 2 studies using different methods, allowing for assessment of the effect of study design. The 2 cross-sectional studies performed in adults in NSW in the postpandemic phase (performed in different laboratories) were included in a regression model that examined the effect of using serum specimens collected to identify pathologic agents from outpatients by comparing them to results with plasma units of donated blood. The 2 collections obtained from WA adults in the postpandemic phase were analyzed similarly to examine the effect of using volunteers in a randomized-controlled trial by comparing those results to results using plasma units of donated blood. Neither the use of opportunistically collected blood specimens nor the use of adults enrolled in randomized controlled trials showed a significant difference on the outcome of seropositivity from results using plasma units of donated blood (Table 5).

**Table 5:** Multivariate logistic regression models comparing specific collections on outcome of seropositivity, with exposures of region, age group, and sex, in community-based studies of influenza A (H1N1) 2009 in the Southern Hemisphere, winter 2009*

Collections compared		No. assays included	Characteristics of model			ORs (95% CIs) for exposure variables		
Comp.	Ref.		Restrictions to inclusion	Rationale		Male sex	Age†	Comp. group/study compared with ref. group/study
K	N	493	Residence in NSW; post; age 16–78 y	Stored pathology specimens survey vs. survey of blood donors (NSW)		0.98 (0.65–1.49); p = 0.93	0.74 (0.66–0.84); p<0.001	1.37 (0.89–2.09); p = 0.15
R	N	204	Residence in WA; post	Patients voluntarily enrolled in RCT vs. blood donors (WA)		1.05 (0.56–1.98); p = 0.88	1.06 (0.86–1.31) p = 0.56	1.48 (0.79–2.79); p = 0.22
D	K	278	Pre; age ≥58 y	Persons in res. care vs. community control group (NSW)		0.49 (0.31–0.79); p = 0.003	2.79 (2.01–3.86); p<0.001	0.34 (0.15–0.79); p = 0.01
M	K	278	Post; age 19–77 y	Persons with HIV infection vs. community control group (NSW)		1.43 (0.80–2.57); p = 0.23	0.74 (0.61–0.90); p = 0.003	1.26 (0.66–2.41); p = 0.48
Q	K	192	Post; age 43–88 y	Hemo. patients vs. community control group (NSW)		0.90 (0.42–1.95); p = 0.79	0.91 (0.68–1.21); p = 0.50	1.65 (0.75–3.63); p = 0.21
J	N, R	316	Res. in WA; post; age 21–45 y	Preg. women vs. community control group (WA)		.	0.72 (0.48–1.06); p = 0.10	0.44 (0.24–0.81); p = 0.008
C	B	1,316	Post; age >21	HCWs vs. community control group (NZ)		0.92 (0.70–1.22); p = 0.56	0.95 (0.88–1.03); p = 0.26	1.09 (0.83–1.42); p = 0.54
F	E	1,080	Post	HCWs vs. community control group (Sing.)		1.12 (0.74–1.71); p = 0.59	0.78 (0.66–0.93); p = 0.006	0.65 (0.41–1.01); p = 0.06
H	E	996	Post; age 21–62	Military personnel vs. community control group (Sing.)		1.19 (0.75–1.88); p = 0.45	0.71 (0.58 – 0.85); p<0.001	0.97 (0.58–1.60); p = 0.89
G	E	858	Post	Res. care group vs. community control group (Sing.)		1.38 (0.89–2.16); p = 0.15	0.81 (0.68–0.96); p= 0.02	0.44 (0.22–0.90); p = 0.03
P	P	1,689	Post	Aboriginal and Torres Strait Islanders vs. nonindig. people (NT)		0.95 (0.74–1.22); p = 0.68	0.88 (0.82–0.94); p<0.001	2.67 (2.08–3.42); p<0.001
B	B	1,147	Post	Maori vs nonindig. people (NZ)		0.95 (0.73–1.22); p = 0.66	0.86 (0.82–0.91); p <0.001	1.17 (0.83–1.64); p = 0.38
B	B	966	Post	Pacific Peoples vs. nonindig. people (NZ)		1.04 (0.78–1.37); p = 0.80	0.87 (0.82–0.92);p<0.001	1.99 (1.41–2.82); p<0.001

*ORs, odds ratios; comp., comparison; ref., referent; NSW, New South Wales; post, postpandemic phase; WA, Western Australia; RCT, randomized controlled trial; pre, prepandemic phase; res., residence/residential; hemo., hemodialysis; preg., pregnant; HCWs, health care workers; NZ, New Zealand; NT, Northern Territory; nonindig., nonindigenous. The 13 regression models are displayed horizontally. †Age considered a continuous variable with OR for each decade of increasing age.

We compared several other pairs of datasets by logistic regression to examine the effect of specific risk factors on the outcome of seropositivity. The odds ratio for the binary variable of study of origin (comparison study vs. reference study) is displayed as an estimate of the effect of the risk factor on the outcome. In NSW during the prepandemic phase, living in a residential care facility was associated with lower levels of preexisting seropositivity than was living in the general community. However, in the postpandemic phase, we found that persons with HIV infection or those who were undergoing hemodialysis were not significantly more likely to be seropositive than were community control subjects. In WA in the postpandemic phase, pregnancy was associated with lower levels of seropositivity. Health care workers in NZ had levels of postpandemic seropositivity similar to community controls, but those of health care workers in Singapore were lower. In Singapore, military personnel had similar levels of postpandemic seropositivity, while staff and residents of residential care facilities had lower levels compared to community controls. Aboriginal and Torres Strait Islander residents of the NT had higher levels of postpandemic immunity than other ethnic groups, as did Pacific Peoples of NZ (Table 5).

## Discussion

We obtained estimates of the full epidemiologic effects of A(H1N1)pdm09 in the 2009 Southern Hemisphere winter by pooling data from several serologic studies performed across our region. We believe that population-based serologic studies give a more direct measure of community exposure to the virus than notification-based data, which are inherently limited by the proportion of cases of infection that are captured by the notification system. The individual-level data enabled us to apply consistent statistical methods across studies. This enabled estimates of seropositivity to be made across more directly comparable groups, as well as assessments of the effects of specific risk factors on seropositivity.

Our community-based, age-standardized estimates of prepandemic seropositive proportions ranged from 3.5% to 11.9%, with Singapore demonstrating a lower level of prepandemic immunity than Australia and NZ. The increased levels of prepandemic immunity in those >75 years of age are likely to be partially due to cross-reacting antibody responses to influenza A/South Carolina/1/1918 and related viruses that were circulating in the early 20th century (*20*). However, the steady increase in seropositivity with age across age groups suggests more recent circulation of influenza viruses with the potential to elicit cross-reacting antibody responses (*21*).

The finding of peak postpandemic seropositivity in the 5- to 14-year age group is consistent with greater social mixing of school-aged children, lower prepandemic immunity, and results from other population-wide studies (*22**,**23*). Despite the low level of prepandemic cross-reactive antibodies to the virus, Singapore remained the region with the lowest proportion of population-standardized seropositivity in the postpandemic phase (17.5%), whereas estimates from Australia and NZ ranged from 22.1% to 32.8%. The implication of lower postpandemic seropositivity in more tropical regions is consistent with estimates from India and Hong Kong (*23*,*24*), as well as with the nonsignificant trend toward greater seropositivity in the more northerly Australian regions (NT and Queensland) on logistic regression. Although overall attack rates were similar for Australia and Singapore, this finding highlights the geographic heterogeneity of influenza spread and suggests that latitude may be a critical predictor of susceptibility to influenza, which might be explained by increased efficiency of transmission of influenza in cold temperatures (*25*) or population levels of vitamin D stores (*26*). The negative overall attack rate in those >75 years of age may have been an anomaly, because this age group had the smallest number of specimens and the prepandemic specimens were predominantly from NSW, while the postpandemic specimens were a combination of specimens from NSW, NZ, and NT. Other age groups had more similar compositions between pre- and postpandemic phases and so are likely to be more directly comparable. Alternative explanations include waning immunity in elderly persons in the months after seasonal influenza vaccination or limitations of study K, which observed the greatest decrease in titers in this age group.

Although several coexisting conditions have been found to be associated with severity of infection with A(H1N1)pdm09, most laboratory-confirmed cases across the Southern Hemisphere have occurred in persons without known risk factors (*27*). We found no increase in risk for postpandemic seropositivity among hemodialysis patients and a group of persons with generally well-controlled HIV infection. These results are consistent with the observation that HIV-infected patients admitted to a hospital for influenza have similar clinical outcomes as do non-HIV patients (*28*). Pregnant women represented ≈7%–9% of patients with laboratory-confirmed cases, severe infections, and admissions to intensive care units in the Southern Hemisphere (*27*). Although it has been postulated that this occurred because of the patients’ younger ages and close contact with children, our results suggest that pregnancy is associated with a lower likelihood of infection, possibly because pregnant women actively avoid infection. Therefore, pregnant women appear more susceptible to severe illness with A(H1N1)pdm09 infection, which may relate to lower levels of immunoglobulin G_2_ (*29*). By contrast, our results suggest that the 6- to 7-fold higher rates of hospitalization seen in indigenous persons in our region are likely to be partially attributable to a higher attack rate in Australia (*30*). We did not find evidence for a higher attack rate among health care workers or military personnel, with levels of seropositivity comparable to those of the general community. A study in a Finnish garrison found that 22.3% of personnel had titers of >40, a level lower than the 33.9% observed in Singaporean military personnel postpandemic, even though this study was performed in response to a recent outbreak (*31*). Levels of seropositivity in those living in residential care appeared lower than in community-living persons of comparable age, both in a prepandemic comparison in NSW and in a postpandemic comparison in Singapore.

The unavoidable limitation to our comparisons is that they included data from multiple studies that used differing methods. Studies differed by epidemiologic approach, specimen type, and laboratory methods, and the jurisdictions studied exhibited different public health responses. We excluded from analysis data we considered to have been obtained with methods that were unlikely to give a population-wide estimate of serologic immunity, for example, 1 retrospective prepandemic collection from persons with postpandemic seropositivity (M) and the postintervention assessments from clinical trials (L, O, R, S). Several studies used convenience collections of specimens taken for clinical indications before routine discarding. These studies enabled population-based estimates but were subject to selection bias, given that conditions predisposing to influenza might increase the chance of being tested. By contrast, the use of blood donor specimens may select for a healthier sample. Cohort studies (E–H) were analyzed in the same manner as for cross-sectional surveys, although samples included in these datasets were determined by selection biases relating to original enrollment in the cohort as well as to enrollees dropping out. Previous evidence indicates that this is a valid approach to estimating population-wide immunity (*32*). Moreover, our analysis found no effect from differing study methods when comparing 2 pairs of studies performed in the same populations. Therefore, while the differences seen between the risk groups could have been caused by differences in study method, we found no evidence of this from the data available.

Whether the epidemiologic differences are due to differences in transmission in differing populations or because of the effectiveness of public health responses is difficult to gauge. In Australia, most jurisdictions moved from the Delay to the Contain phase on May 22 and from the Contain to the Protect phase on June 22. Only Victoria, which contributed 234 specimens to this pooled analysis, differed in the timing of its response phases (*33*). Although there were notable differences between public health management of the response to the outbreak in NZ and Singapore, the timing of the transition to containment was broadly similar, with NZ focusing primarily on containment from April 25 to June 21 (*34*), while Singapore began its transition to the Mitigation phase on June 29 (*35*).

Protocols for the HI assay may differ between laboratories in terms of specimen source and preparation (serum or plasma, erythrocyte adsorption), reagents (erythrocyte species, antigen preparation), procedure (incubation conditions), and controls. Furthermore, use of fresh erythrocytes for HI assays means inherent within-laboratory variability must be managed. To minimize variability between laboratory method and erythrocyte batches, control panels of serum samples were shared and results were standardized. A common source of virus antigen was also shared. Such comparative experiments were performed early in the pandemic between 3 of the 4 laboratories described in this analysis, with minimal variation seen. These 3 laboratories used a common source of A(H1N1)pdm09 antigen for at least 15 of the 19 datasets included. International standards were also available in 2009 for standardization of serologic assays around the world. Notably, the source of erythrocytes to detect influenza virus may vary, depending on the binding specificity of the hemagglutinin protein for each virus. A(H1N1)pdm09 virus recognized human, turkey, and guinea pig erythrocytes. This enabled laboratories to use cells that were available and that they were experienced in handling.

Although all studies used a titer of >40 as the cutoff for seropositivity, this is an over-simplification of the complex immune response to influenza infection, which includes both cellular and humoral components (*36*). Although a titer of 40 was achieved in 80%–90% of persons with PCR-confirmed infection with A(H1N1)pdm09 (*37*), in unpaired analysis, no single cutoff reliably determines past infection and subsequent immunity. Serologic studies that incorporated interviewing participants about symptoms of influenza-like illness suggest that as many as half of those with serum titers of >40 in the postpandemic phase do not have a history of a compatible illness (*16**,**31*). This finding partly reflects the fact that a proportion of those patients who were seropositive in the postpandemic phase were already seropositive in the prepandemic phase, but also suggests that some persons who seroconverted did not experience or report symptoms. Despite this, pre- and postpandemic cross-sectional serologic surveys are the most convenient and inclusive method for assessing population-wide serologic immunity.

Our results provide a broad picture of the effects of A(H1N1)pdm09 in the Southern Hemisphere during the winter of 2009. The absence of clear differences between estimates with different study methods suggests that pooling of data is likely to be useful in estimating the effects of the virus across population groups. We found greater levels of prepandemic seropositivity as patient’s age increased, particularly in those >75 years of age. By contrast, in the postpandemic period, school-aged children showed the greatest levels of immunity. Health care workers, military personnel, persons with HIV infection, and hemodialysis patients had levels of postpandemic seropositivity similar to those of the general community. Pregnancy and residential care appeared protective from infection, suggesting more severe disease in those infected. Despite recording the lowest prepandemic levels of immunity, Singapore retained comparatively lower levels of seropositivity after the pandemic.

Technical Appendix Provides dates samples collected in serologic studies and dates used to define pandemic phases in serologic studies.
