# Supplementary material for: Seroepidemiologic Effects of Influenza A(H1N1)pdm09 in Australia, New Zealand, and Singapore
Source: Emerg Infect Dis. 2013 Jan;19(1):92–101. doi: 10.3201/eid1901.111643 (PMC3557971; doi:10.3201/eid1901.111643)
Supplement: Technical Appendix — Provides dates samples collected in serologic studies and dates used to define pandemic phases in serologic studies. [file 11-1643-Techapp-s1.pdf]

Article DOI: http://dx.doi.org/10.3201/eid1901.111643

Seroepidemiologic Effects of Influenza A(H1N1)pdm09 in Australia, New Zealand, and Singapore

Technical Appendix

Technical Appendix Table 1. Dates samples collected in serologic studies to estimate attack rates of influenza A (H1N1) pandemic 2009 in the Southern Hemisphere, winter 2009.

Study	Start date	End date
A	2004 Apr 14	2009 Apr 22
B	2009 Nov 12	2010 Apr 13
C	2009 Dec 21	2010 Mar 4
D	2006 Oct 7	2009 Jul 16
E	2005 Jun 29	2009 Jun 3
	2009 Jun 20	2009 Jun 27
	2009 Aug 20	2009 Aug 29
	2009 Oct 6	2009 Oct 11
F	2009 Jun 22	2009 Jul 7
	2009 Aug 19	2009 Sep 3
	2009 Sep 23	2009 Oct 15
G	2009 Jul 17	2009 Jul 28
	2009 Oct 5	2009 Oct 7
H	2009 Jun	2009 Jul 1
	2009 Aug 20	2009 Sep 3
	2009 Sep 10	2009 Oct 9
I	2008 Nov 3	2009 May 15
	2009 Aug 1	2009 Nov 30
J	2009 Jan 2	2009 Feb 27
	2009 Aug 2	2009 Sep 30
K	2007 Jul 3	2008 Dec 30
	2009 Aug 3	2009 Sep 30
L	2009 Jul 22	2009 Jul 26
M	2009 Jun 1	2009 Sep 29
N	2009 Apr 2	2009 May 20
	2009 Oct 13	2009 Oct 30
	2009 Nov 16	2009 Dec 1
O	2009 Aug 3	2009 Sep 4
P	2009 Jan 10	2009 May 29
	2009 Sep 3	2009 Sep 30
Q	2009 Nov 10	2009 Nov 25
R	2009 Apr 19	2010 Jan 25
S	2008 Sep 1	2009 Jun 16
	2009 Sep 1	2010 Jun 2

Technical Appendix 2. Dates defining pandemic phases in serologic studies to estimate attack rates of influenza A (H1N1) pandemic 2009 in the Southern Hemisphere, winter 2009

Region	First notified case	90% of 2009 cases notified + 2 weeks
New South Wales	May 20	Aug 24
New Zealand	Apr 25	Jul 31
Northern Territory	May 29	Aug 30
Queensland	May 8	Aug 31
Singapore	May 26	Not defined
South Australia	May 22	Sep 14
Tasmania	May 21	Aug 23
Victoria	May 20	Aug 15
Western Australia	May 24	Sep 7
